# PCR amplification of a triple-repeat genetic target directly from whole blood in 15 minutes as a proof-of-principle PCR study for direct sample analysis for a clinically relevant target

**DOI:** 10.1186/s12881-014-0130-5

**Published:** 2014-12-12

**Authors:** Christopher M Connelly, Laura R Porter, Joel R TerMaat

**Affiliations:** Streck, Inc., 7002 S. 109th Street, LaVista, Omaha, NE 68128 USA

**Keywords:** Direct-PCR, Rapid-PCR, Direct-Blood PCR, Myotonic dystrophy, Triple-repeat, Philisa, Streck, Genetic screen

## Abstract

**Background:**

Most PCR-based diagnostics are still considered time- and labor-intensive due to disparate purification, amplification, and detection steps. Advancements in PCR enzymes and buffer chemistry have increased inhibitor tolerance, facilitating PCR directly from crude samples. Obviating the need for DNA purification, while lacking a concentration step, these direct sample methods are particularly apt for human genetic testing. However, direct PCR protocols have traditionally employed thermal cyclers with slow ramp rates and conservative hold times that significantly increase an assay’s time-to-result. For this proof-of-principle study, our objective was to significantly reduce sample preparation and assay time for a PCR-based genetic test, for myotonic dystrophy type 1 (DM1), by pairing an inhibitor-resistant enzyme mix with a rapid thermal cycler to analyze samples directly in whole blood.

**Methods:**

DM1 genetic screening was done with an adapted conventional PCR approach that employed the Streck Philisa® Thermal Cycler, the inhibitor-resistant NEBNext® High-Fidelity 2X PCR Master Mix, and agarose gel electrophoresis or an Agilent 2100 Bioanalyzer for detection. The Gene Link™ Myotonic Dystrophy Genemer™ Kit was used as a reference assay kit to evaluate the rapid assay.

**Results:**

In this work, a rapid and direct PCR assay testing 10% whole blood as template has been developed as an exclusionary screening assay for DM1, a triple-repeat genetic disorder. PCR amplification was completed in 15 minutes using 30 cycles, including *in situ* hot-start/cell lysis. Out of the 40 donors screened, this assay identified 23 (57.5%) as DM1 negative suggesting no need for further testing. These data are 100% concordant with data collected using the commercially available Gene Link Genemer™ Kit per the kit-specific PCR protocol.

**Conclusions:**

The PCR assay described in this study amplified DM1 short tandem repeats in 15 minutes. By eliminating sample purification and slower conventional PCR protocols, we demonstrated how adaptation of current PCR technology and chemistries can produce a simple-to-use exclusionary screening assay that is independent of up-front sample prep, improving a clinical lab technician’s time-to-result. We envision this direct and rapid methodology could be applied to other conventional PCR-based genetic tests and sample matrices where genomic DNA is targeted for analysis within a given molecular diagnostic platform.

## Background

PCR-based testing, using whole blood as the DNA source, is increasingly being used for forensic analysis, diagnosis of microbial infection, detection of genetic disease, screening of organ transplants, blood banking, and prenatal diagnosis [1-7]. Improvements to enzymes, specifically hot-start formulations and recombinant engineering modifications [8], have promoted adoption of PCR by increasing assay robustness and convenience of reaction preparation. However, a critical limitation of PCR analysis is the time-consuming and costly DNA extraction steps often required for successful amplification of DNA derived from clinical samples. Even with extraction, PCR inhibitors can remain problematic and methods to overcome false-negatives or reduced sensitivity must be carefully selected to ensure the integrity of acquired data [9-12]. Consequently, PCR enzymes and buffer chemistry have been designed to amplify target sequences directly from complex inhibitor-laden matrices such as whole blood, soil, feces, and plants [13,14]. When working with whole blood, common inhibitors of PCR include: hemoglobin/heme, immunoglobulin G, lactoferrin, myoglobin, and blood collection tube chemicals (i.e., EDTA, heparin, and sodium citrate) [15].

The feasibility of such direct sample PCR techniques in complex matrix backgrounds, in part, can be attributed to the directed evolution of DNA polymerases. For example, the engineering of chimeric enzymes, which combine polymerase and protein-specific DNA binding domains, have yielded polymerases with increased processivity without compromising activity or stability [16]. Use of site-directed mutagenesis has produced recombinant polymerases demonstrating a high resistance to inhibitors [13,14,17]. Chemical additives and enzyme blends have also provided a mechanism to overcome PCR inhibitors and facilitate DNA amplification—even in the presence of unpurified samples or templates with high GC content [13]. Because each PCR inhibitor’s mode of action can vary, Barnes and coworkers demonstrated that a cocktail of common additives (betaine, trehalose, L-carnitine, and NP-40) improved PCR amplification from complex sample matrices [13]. Furthermore, the addition of these PCR enhancer cocktails and an inhibition-resistant polymerase to complex samples generally results in successful PCR amplification.

In addition to inhibition-resistant buffers and polymerases, one must consider the thermal cycling protocol needed to implement a rapid direct-PCR assay. While the reaction chemistries are fairly established, most PCR ramp rates and hold times in direct sample assays have traditionally been conservative to achieve the desired amplification of target, especially by increasing the extension time to allow the enzyme to persevere over inhibitors. Appropriate pairing of direct sample PCR with fast thermal cycling and/or detection holds promise for unprecedented time-to-results. Recently, Aboud and colleagues demonstrated <25-minute forensic genotyping directly from FTA buccal swabs using ultra-fast thermal cycling and a prototype Agilent bioanalyzer [18]. However, to our knowledge, there have been no reports detailing the combination of rapid cycling technology with a more technically challenging and inhibitor-laden matrix such as whole blood. Adding further challenge to direct sample methods, direct PCR methods do not have a DNA concentration step; therefore, a larger PCR sample volume is advantageous to facilitate maximal template-to-volume input. The Streck Philisa® Thermal Cycler, due to the nature of the heat transfer and sample tube design, maintains high ramp rates and sample homogeneity with sample volumes up to 50 μl.

In this work, we describe a rapid and direct blood PCR assay as a proof-of-principle to demonstrate amplification of short tandem-repeat (STR) sequences designed to detect myotonic dystrophy type 1 (DM1) alleles. DM1 is an autosomal dominant disorder and is the most common form of adult onset muscular dystrophy with an incidence of about 1 in 8,000 individuals [19]. DM1 is characterized by a CTG triple-repeat expansion in the 3′-untranslated region of the *DMPK* gene [20]. Because severity of the disease correlates with the degree of repeat expansion, detection of “normal” sized alleles has previously been used to exclude samples with DM1-negative genotypes [21]. Guidelines for genetic testing of DM1 indicate the number of CTG repeats range from 5 to 34 for a normal allele [21]. Repetitions of 35 to 49 are considered pre-mutations; carriers are generally asymptomatic, but genomic instability of these repeats can put offspring at increased risk for inheriting a larger allelic repeat size. When repeat lengths are greater than 50, persons are often symptomatic and severity usually correlates with increasing repeat length. For diagnosis, conventional PCR can be used as the first step in DM1 testing [19]. However, the test is most beneficial when two normal size alleles are identified; in this case, DM1 can be excluded and secondary methods of analysis are not required [21]. On average, ~25% of disease-negative population is homozygous with a normal allele, which cannot be discriminated by conventional PCR assays. Therefore, the presence of a single DM1 allele does not confirm DM1 genotypes associated with disease phenotype. These samples require follow-up testing with triple-repeat PCR or Southern blotting methodology [21].

The purpose of this study was to demonstrate successful PCR amplification of these genetic targets in 15 minutes for samples containing up to 30% whole blood. Furthermore, a 30-cycle 15-minute DM1 PCR directly from 10% crude whole blood was an optimal method to demonstrate feasibility of a rapid direct PCR-based approach for detection of genomic DNA targets. Blood samples from 40 donors were screened for the absence of DM1-associated allelic expansions. These data demonstrate a synergistic coupling of a fast PCR cycling strategy and direct blood PCR. The method is broadly applicable and could be optimized with alternative genetic targets and sample matrices. Although rapid and direct PCR methods are still a work in progress, assays such as this demonstrate promise for clinical use by decreasing sample handling and expediting analysis to reduce a molecular assay’s time-to-result, providing a more cost-effective solution for genetic analysis in the clinic.

## Methods

### Blood donor recruitment and blood collection

This study was approved by the institutional review board of the Methodist Hospital (Omaha, NE, USA) and informed consent was obtained from all donors. All blood donors were anonymous volunteers recruited from Streck (Omaha, NE, USA). Both male and female donors were tested and presumed to be healthy. A 10 ml blood sample was drawn by venipuncture into a K_2_EDTA blood collection tube (BD Vacutainer®, Becton Dickinson, Franklin Lakes, NJ U.S.A.) for each donor. Blood was mixed well immediately after the draw by inverting the tube 10 times.

### Extracted DNA samples

Blood samples for direct addition to PCR sample mixes were frozen and stored at −80 °C until all aliquots were acquired for analysis. Thawed blood was used for direct PCR. For control samples, purified DNA from whole blood was obtained using the AutoGen QuickGene-810 (Catalog No.: FI810; Holliston, MA, USA) with the AutoGen DNA Whole Blood Kit (Catalog No.: FK-DBS).

### PCR Amplification

**Figure 1:**
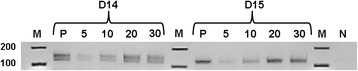
**Investigation of % blood tolerance of the rapid PCR assay.** Increasing concentrations of whole blood (5, 10, 20, or 30%), as indicated, were added to the PCR tube. Products resolved by agarose gel electrophoresis. M (100 bp marker); P (10 ng of purified donor DNA); D14 (Donor 14); D15 (Donor 15); N (no template control).

The primer set for the myotonic dystrophy assay was obtained from GeneLink (Catalog No.: 40-2026-10, Hawthorne, NY USA) and yields a product size of 114 + 3 N bp, where N is the number of triple-repeats. NEBNext® High-Fidelity 2X PCR Master Mix was provided by New England BioLabs (Catalog No.: M0541, Ipswich, MA USA). Each 25 μl reaction volume contained 0.5 μM of each primer and 1X NEBNext® High-Fidelity PCR Master Mix. Purified DNA or whole blood amounts for each reaction were used as described in the text. For Figure 1, 3 mM MgCl_2_ was required for amplification of the DM1 alleles from 30% whole blood. The standard 1X mix has 2 mM MgCl_2_, and increasing this to 3 mM did not improve amplification for other blood concentrations tested in this study. All reactions used MB grade nuclease-free water (Sigma, Catalog No.: W4502, St. Louis, MO USA). A 2-step PCR protocol was carried out using the Streck Philisa® Thermal Cycler (Catalog No.: 250000, Omaha, NE USA) and Philisa PCR Tubes (Catalog No.: 250005): Hot start of 98 °C for 3 minutes, followed by 30 cycles of [98 °C for 6 seconds and 68 °C for 12 seconds]. As a procedural control the Gene Link™ Myotonic Dystrophy Genemer™ kit (Catalog No.: 40-2026-11, Hawthorne, NY USA) was used as per manufacturer’s instructions, except Platinum® Taq DNA Polymerase (Life Technologies, Catalog No.: 10966-026, Grand Island, NY USA) was added to the PCR mix prior to the hot-start.

### Analysis of PCR products

PCR products retained in the supernatant (after a 20-second pulse on a picofuge to separate out cellular debris) were resolved by agarose gel electrophoresis and an Agilent 2100 Bioanalyzer (Agilent Technologies, Loveland, CO USA). For gel detection, 10 μl of PCR product was stained with ethidium bromide and resolved on a 3% agarose gel using TAE buffer. Imaging of ethidium bromide-stained PCR product was done on a Molecular Imager VersaDoc™ (Bio-Rad; Hercules, CA USA). Bioanalyzer sample analysis was carried out using the Agilent 1000 DNA kit (Catalog No.: 5067–1504) as per manufacturer’s specifications.

## Results and discussion

To demonstrate the utility and reliability of a rapid and direct blood PCR approach, we used whole blood to screen presumed healthy donors for the presence of normal DM1 alleles. As a comparative control, results were compared to data collected using each donor’s purified DNA (pDNA) and a commercially available kit for DM1. In the *DMPK* gene, 5 to 34 triple-repeats (normal alleles) correspond to a DNA fragment size of approximately 129 to 216 bp (114 + 3 N), an amplicon size readily detected with conventional PCR. Although PCR detection of expanded (or even pre-mutation) alleles is ideal, our focus with this assay was identification of *DMPK* alleles clearly under the 216 bp cut-off. In this case, it is feasible that a simple conventional PCR test can function as a negative screen for exclusion of DM1 heterozygotes within the normal base pair range, i.e., when amplicon length clearly indicates alleles of normal size [21]. If two normal alleles are identified, DM1 can be excluded and secondary methods of analysis are generally not required. However, since heterozygote frequency for the CTG repeats is ~75% in the normal population, ~25% of unaffected individuals will be homozygous for a given normal allele [21]. Therefore, the presence of a single PCR band or a PCR band bordering the molecular size threshold of normal and pre-mutation is not meant to confirm a diagnosis of DM1. Alternatively, these samples would require more quantitative testing, such as Southern blot or triple-repeat PCR [21], to determine the presence of expanded alleles. In compliance with current guidelines for reporting only the presence of normal, pre-mutation, or expanded alleles, exact repeat sizing for the assay described here is not a necessity if both heterozygous alleles fall within the normal molecular size range and are detected with a standard PCR assay [21]. However, the exact allele size is imperative when conventional methods produce unclear data that does not discriminate an expanded pre-mutation or fully penetrant allele. Pre-mutation alleles are not associated with a clinical phenotype in the carrier but can expand in future generations; however, fully penetrant alleles are associated with a disease phenotype [21].

To determine if a rapid PCR assay could detect DM1 normal alleles directly from blood, we tested assay tolerance with increasing blood concentrations for two different donor samples using multiple polymerases and master mixes (data not shown). During enzyme and buffer screening, most enzymes advertised for direct PCR analysis were not compatible with the shortened hold times favored in rapid PCR protocols. Conversely, many high-fidelity polymerases that successfully amplified target from pure DNA samples, using the Philisa 15-minute cycling protocol, did not amplify target when whole blood was added directly to the master mix. For the purposes of our study, the NEBNext High-Fidelity 2X PCR Master Mix was used because it produced PCR product successfully for both rapid cycling and direct-blood analysis when paired with the Philisa Thermal Cycler. Data from Figure 1 indicate the assay could tolerate up to 30% whole blood in a given PCR reaction tube and the cycling time was completed in 15 minutes, including a 3-minute hot-start/cell lysis step. Some qualitative loss of band intensity is observed during testing of whole blood concentrations at 30%, which likely results from increased inhibitor concentration scaled alongside increased template input. Increasing MgCl_2_ concentration in the reaction buffer by 1 mM improved amplicon resolution on the gel for samples tested at 30% whole blood. Donors 14 and 15 in Figure 1 provide an example of heterozygous and homozygous DM1 alleles, respectively. Based on Figure 1 data for donor 14, PCR using DM1-specific primers detected two amplicons that clearly resolve below the 216 bp cut-offs for normal allele sizing and, as such, is an example of a DM1 negative genotype. However, donor 15 would require further testing to determine if the patient carries a higher molecular weight repeat expansion or is homozygous for the normal allele. Data from Figure 1 demonstrates the application of this test as a rapid exclusionary screening assay for DM1.

**Figure 2:**
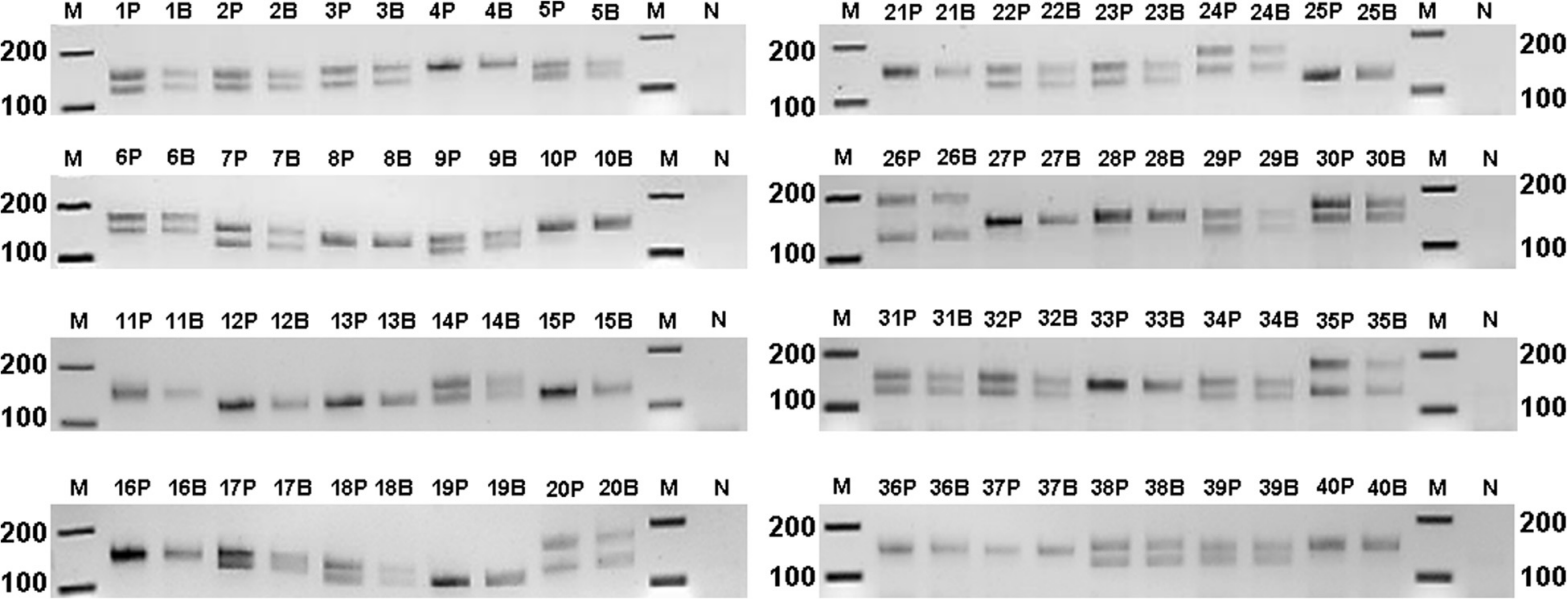
**Rapid PCR screening of DM1 with agarose gel detection.** 10 ng purified donor DNA (P) or 10% direct whole blood (B) from 40 numerically-indicated donors with detection using agarose gel electrophoresis. N (no template control); M (marker; the 200 to 100 base pair range is indicated).

**Figure 3:**
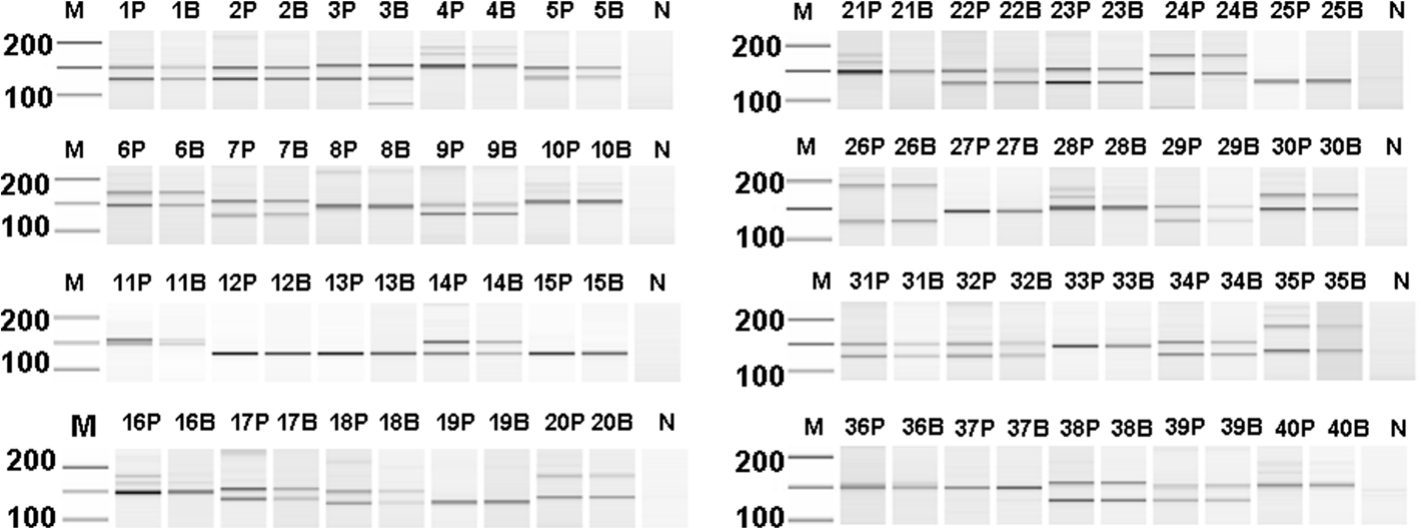
**Rapid PCR screening of DM1 with Agilent bioanalyzer detection.** 10 ng purified donor DNA (P) or 10% direct whole blood (B) from 40 numerically-indicated donors with detection using the Agilent 2100 Bioanalyzer DNA 1000 kit. N (no template control); M (marker; the 200 to 100 base pair range is indicated).

Using the same 15-minute PCR assay with optimized whole blood concentration set at 10%, we increased our sample set to 40 donors to test the reliability of detection methods by using both agarose gels (Figure 2) and an Agilent bioanalyzer (Figure 3). Gel data, concordant between purified DNA and whole blood, identified 23 of the 40 donors as negative for DM1; this is indicated by the presence of two molecular weight bands between ~129 to 216 bp and clearly under the 216 bp normal allele cut-off. As such, 17 of the 40 donors would require further testing to determine their disease status. Of the donors tested, 57.5% of our sample set were heterozygous for the DM1 allele and would have been identified as negative for DM1: a value 20% lower than the global population average.

**Figure 4:**
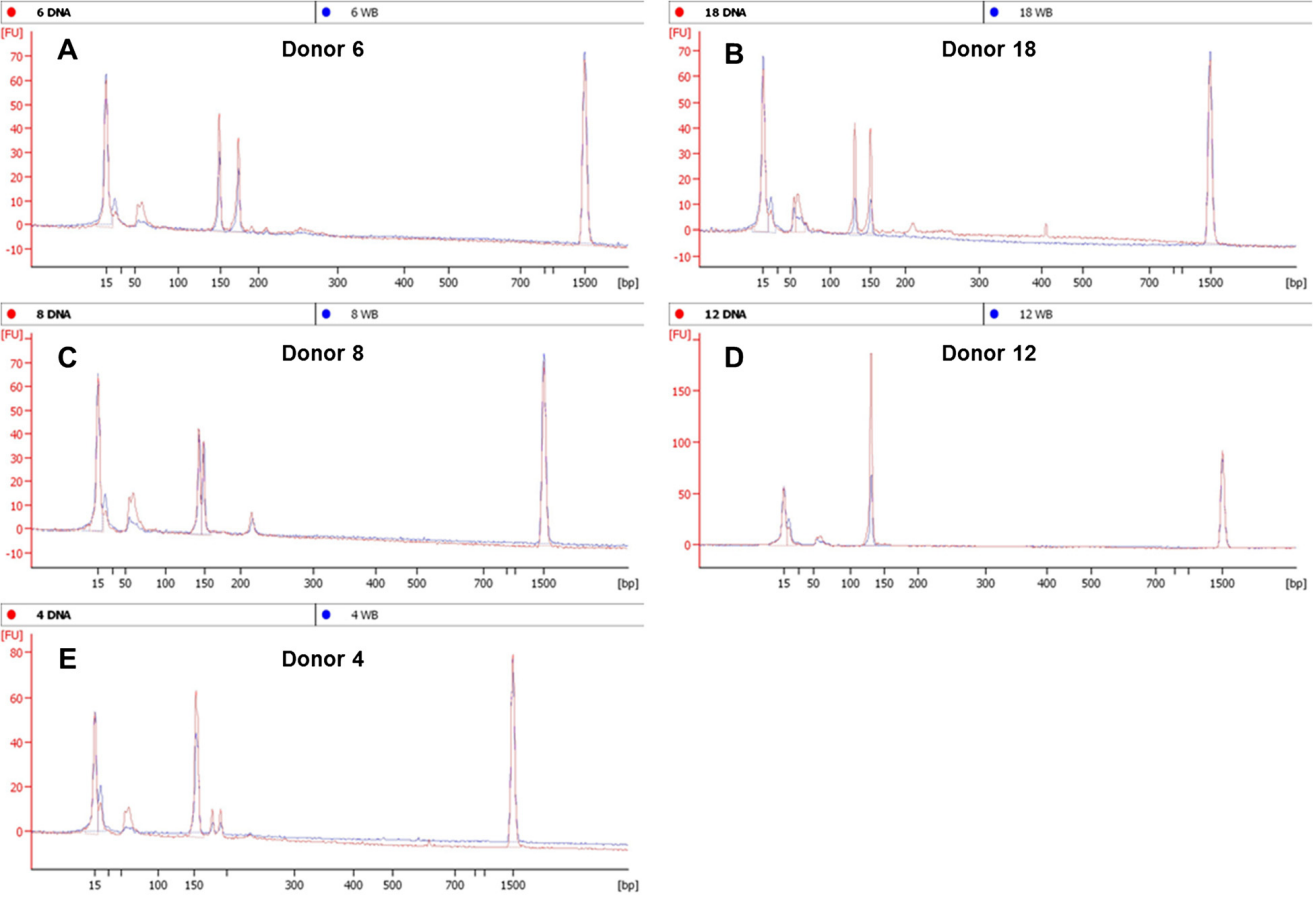
**Selected bioanalyzer electropherogram overlays from purified DNA (red) or 10% whole blood (blue).** Results from five representative donors illustrate examples of a confirmed heterozyote **(A)**, heterozygote with a 10% blood under the default FU cutoff **(B)**, and suspected heterozygote with split peaks as an allelic differentiator and a larger artifact **(C)**, confirmed homozygote **(D)**, homozygote with two smaller artifacts **(E)**.

To this end, we investigated whether the improved detection sensitivity of an Agilent bioanalyzer could reveal any additional heterozygous donor alleles that were not readily resolved on an agarose gel. The bioanalyzer data (both purified DNA and whole blood results) did strongly support gel data for the 23 donors detected as heterozygotes and DM1-negative as well as the 17 donors that needed further testing (Figure 2). However, for three donors (8, 11, and 36; Table 1, Figures 3 and 4) characterized as homozygotes by gel electrophoresis data, the bioanalyzer produced split peaks. Based on our manual interpretation of the data and the resolution sensitivity of the assays, it is possible that the DM1 alleles for these donors are a CTG repeat apart (Figure 4C); however, this is a size difference outside the resolution limits of both an agarose gel and the bioanalyzer. For example, the DNA 1000 kit user manual indicates a 200 bp fragment detected with the Agilent bioanalzyer has a 5% error when resolving 100 to 500 bp amplicons, a resolution too low to resolve a single CTG repeat. Alternatively, we cannot rule out that the split peaks are a product of incomplete amplicon extension during the PCR. In these cases, the results are questionable and should be interpreted as in need of further testing. In agreement with agarose gel data, the heterozygous donors detected with the Agilent bioanalyzer are still below the global heterozygous frequency. These differences could be attributed to (1) the very small and localized population sample set, (2) decreased size resolution compared to other detection methods, and (3) the inability to identify pre-mutation and expanded alleles.

**Table 1:** **Bioanalyzer data summary for DM1 direct blood PCR results**

**Donor(s)**	**Result**	**Resolution**
1, 2, 3, 5, 6, 7, 9 14, 17, 22, 23, 24, 30, 34, 38, 39	Heterozygote	Both gel and bioanalyzer data indicate bands or peaks, respectively, consistent with two alleles within the specified base pair range for a normal DM1 phenotype. Detected peaks were above the bioanalyzer’s assay 20 FU default threshold for both purified DNA and 10% whole blood. No further testing needed.
18, 20, 26, 29, 31, 32, 35	Heterozygote-Manual Call*	The purified DNA sample and 10% whole blood sample both indicate heterozygote. However, for 10% whole blood samples, agarose gel detection indicated low band intensity and bioanalyzer electropherogram peaks were under the 20 FU default threshold.
8, 11, 36	Gel: Homozygote Bioanalyzer: Heterozygote “Split-Peak”*	The PCR product(s) for this donor sample have an apparent split peak when detected by the Agilent bioanalyzer. Agarose gel data indicate a single band. If reported bioanalzyer resolution is accurate, the split peak would indicate a tandem repeat difference of 5–10 base pairs for these DM1 amplicons.
12, 13, 15, 19, 25, 27, 33, 37	Homozygote*	Both gel and bioanalyzer data indicate bands or peaks, respectively, consistent with one allele within the specified base pair range for a normal DM1 phenotype. Further testing is recommended to determine if there is a DM1 repeat expansion.
4, 10, 16, 21, 28, 40	Homozygote-Artifacts*	Agarose gel data for this sample indicate a single clear band for both purified DNA and 10% whole blood. However, for the more sensitive bioanalyzer studies, smaller peaks corresponding to larger base pair fragments were identified. We speculate these peaks are non-specific PCR amplification products. Peaks detected with the bioanalyzer, falling under the assay’s 20 FU default threshold, were interpreted as artifacts. Further testing would be required to determine DM1 status for this patient.

*Donors with the following results would require further testing based on bioanalzyer data.

Furthermore, in contrast to gel electrophoresis data, the bioanalyzer results required careful interpretation. In some of the samples, spurious peaks and baseline artifacts (Figure 4C and E) arose, likely due to the increased detection sensitivity of the bioanalyzer. For example, in some samples, two smaller peaks around 175 bp (Figure 4E) or a broad peak around 220 bp (Figure 4C) were observed. Gel lanes for no template control (NTC) samples did not resolve any amplicons, which suggests contamination of the PCR master mix was not an issue (Figure 3; see gel lanes marked N). Given that the intensities of these peaks were small relative to clear product peaks, we dismissed them as artifacts. Denoting these as artifacts is considered a valid assumption for samples in which two higher intensity heterozygote peaks are also clearly present. For samples where one clear product peak is present along with questionable peaks, we called these samples as homozygotes (Figure 4D). Regardless of homozygote or questionable call notation, more quantitative tests are necessary to discriminate between artifact, pre-mutation, or a fully penetrant allele. In another aspect, for some whole blood samples the product peaks fell below the default software threshold of 20 fluorescent units (Figure 4B). However, we interpreted these as product peaks by manual calling given the accompanying clean baseline and supporting agarose gel data. Lastly, for some donors, signal was greater for DM1 amplicons in the whole blood amplification vs. purified DNA and *vice versa*. Even with consistent sample mixing, it cannot be assumed that whole blood samples added to each PCR tube in the direct blood assay had equivalent DNA concentration compared to the 10 ng purified DNA controls. Furthermore, PCR product-trapping in red cell debris pellets may result in minor sample loss during the post-PCR centrifugation step. Table 1 provides a summary of all donors that indicates results with the rationale for their assignment following assay screening.

**Figure 5:**
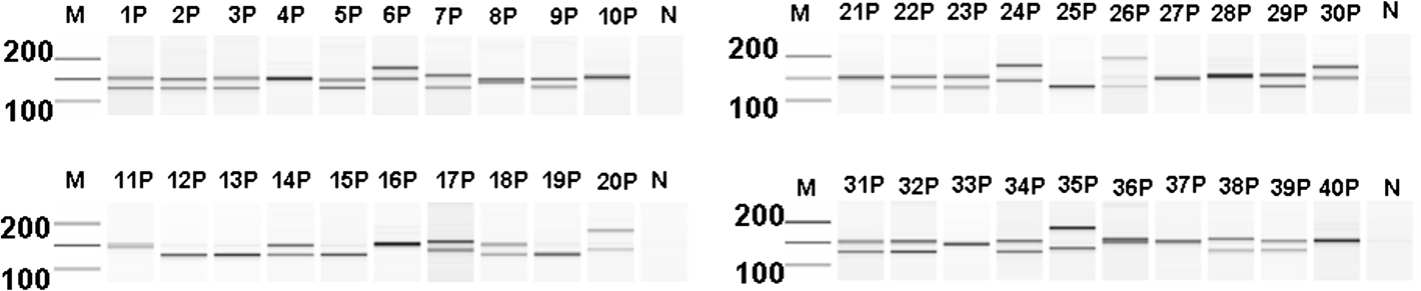
**DM1 screening using the Gene Link Genemer Kit with Agilent bioanalyzer detection.** 100 ng purified donor DNA (P) from 40 numerically-indicated donors with detection using the Agilent 2100 Bioanalyzer DNA 1000 kit. N (no template control); M (marker; the 200 to 100 base pair range is indicated).

**Figure 6:**
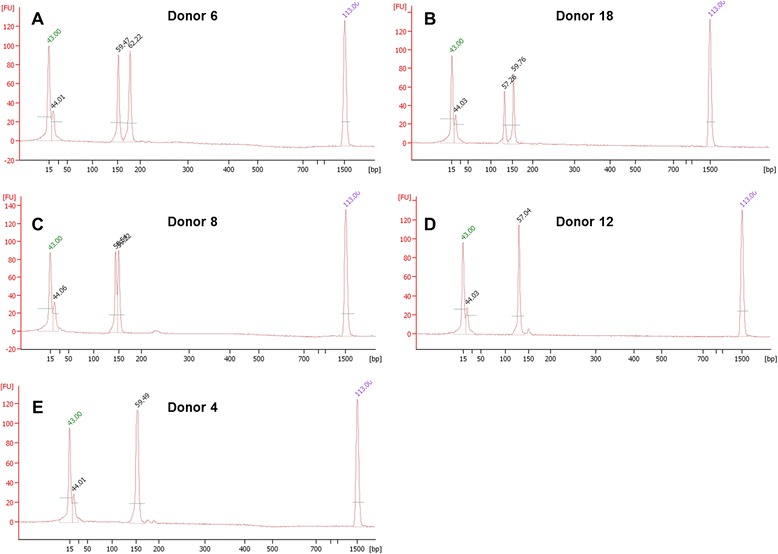
**Selected bioanalyzer electropherogram overlays from purified DNA (red) using the Gene Link Myotonic Dystrophy Genemer Kit.** Results from five representative donors illustrate examples of a confirmed heterozyote **(A)**, heterozygote with a 10% blood under the default FU cutoff **(B)**, and suspected heterozygote with split peaks as an allelic differentiator and a larger artifact **(C)**, confirmed homozygote **(D)**, homozygote with two smaller artifacts **(E)**.

To further substantiate the rapid and direct PCR results described herein, all purified donor DNA was tested using the Gene Link Genemer Kit (Figure 5). The PCR cycling protocol for this kit takes greater than 2 hours of thermal cycler time and utilized 10-fold more DNA per reaction. In support of the results produced using the rapid and direct assay, the data for these experiments suggests the same 23 of 40 donors were heterozygous and had two normal sized DM1 alleles. Electropherograms for this data are also consistent with data collected in Figure 4; additional peaks were noted and similar to those detected with the rapid and direct PCR protocol (Figure 6). Because the samples tested in these experiments were not previously characterized with more quantitative methods, we cannot use the data to draw a clinical conclusion. However, data for Figures 5 and 6 indicate our 15-minute rapid PCR assay produces similar results to a commercially available kit; it supports the proof-of-concept that this assay could be validated for conventional PCR testing of DM1 genetic targets from whole blood samples as an exclusionary screening assay.

In addition, the use of a bioanalyzer provided a rapid post-PCR analysis platform that reduces the ~60-minute run time of an agarose gel to 35 minutes; this makes the total assay time-to-result for this direct blood method 50 minutes. Even though the bioanalyzer does appear to give better heterozygote resolution and approximate (but unnecessary) sizing, the trade-offs are increased cost and peak interpretation to include/exclude actual vs. artifact peaks. As such, we would only recommend using agarose gel data that clearly indicate two amplicons within the prescribed base pair range and proceed with additional testing for all other samples. While time-to-result for myotonic dystrophy screening is not of grave importance, the sheer convenience of the rapid technique is advantageous for lab productivity and fits typical screening of individuals and families. Although the immediate goal of this work was to focus strictly on the rapid direct PCR assay feasibility, future work with the DM1 assay could test known clinical samples in a head-to-head comparison of this method to a “gold standard” assay for call verification; future work would involve pre-mutation and expanded allele validation as well as implementation on genetic analyzer platforms using fluorescently labeled primers. It is expected that larger pre-mutation-sized alleles can be detected with the assay; i.e., an increase to the repeat sizes is likely to be captured, especially with minimally increased cycling extension times.

## Conclusions

In conclusion, the experiments described in this study demonstrate successful amplification of the triple-repeat disorder DM1 directly from whole blood using a rapid 15-minute PCR protocol that includes a hot-start/cell lysis step. The pairing of rapid thermal cycling and direct sample PCR creates a fast and convenient assay. It circumvents laborious DNA purification methods and can be adapted to other, more specific molecular applications and sample matrices, especially where genomic DNA contains the target(s) of interest. For example, depending on the load present in blood, pathogen detection may also be amenable to direct PCR. Since PCR sensitivity can be an issue critical to pathogen detection, a sample enrichment process or higher concentrations of whole blood may be required for this type of analysis. The assay developed herein has significant potential to provide benefit to molecular labs by improving sample turnaround times, expediting time-critical results, and increasing lab productivity.

## Abbreviations

DM1: Myotonic dystrophy type 1
STR: Short tandem repeat
PCR: Polymerase chain reaction
DNA: Deoxyribonucleic acid
pDNA: Purified DNA
NTC: No template control
RBC: Red blood cell
WBC: White blood cell
bp: Base pairs

## References

[1] Bussani C, Cioni R, Mattei A, Fambrini M, Marchionni M, Scarselli G (2007). Prenatal diagnosis of common aneuploidies in transcervical samples using quantitative fluorescent-PCR analysis. Mol Diagn Ther.

[2] Cursons RT, Jeyerajah E, Sleigh JW (1999). The use of polymerase chain reaction to detect septicemia in critically ill patients. Crit Care Med.

[3] Deng Z, Wu G, Li Q, Zhang X, Liang Y, Li D, Gao S, Lan Y (2006). Noninvasive genotyping of 9 Y-chromosome specific STR loci using circulatory fetal DNA in maternal plasma by multiplex PCR. Prenat Diagn.

[4] Espy MJ, Uhl JR, Sloan LM, Buckwalter SP, Jones MF, Vetter EA, Yao JD, Wengenack NL, Rosenblatt JE, Cockerill FR, Smith TF (2006). Real-time PCR in clinical microbiology: applications for routine laboratory testing. Clin Microbiol Rev.

[5] Lo YM, Leung TN, Tein MS, Sargent IL, Zhang J, Lau TK, Haines CJ, Redman CW (1999). Quantitative abnormalities of fetal DNA in maternal serum in preeclampsia. Clin Chem.

[6] Rautenberg P, Lubbert C, Weers W, Boetel E, Schweichler J, Zhou L, Costard-Jackle A, Kraemer-Hansen H, Harder TC (1999). Evaluation of the AmpliSensor PCR and the SHARP signal detection system for the early prediction of symptomatic CMV infection in solid transplant recipients. J Clin Virol.

[7] Robertson JM, Walsh-Weller J (1998). An introduction to PCR primer design and optimization of amplification reactions. Methods Mol Biol.

[8] Kranaster R, Marx A (2010). Engineered DNA polymerases in biotechnology. Chembiochem.

[9] Dauphin LA, Hutchins RJ, Bost LA, Bowen MD (2009). Evaluation of automated and manual commercial DNA extraction methods for recovery of Brucella DNA from suspensions and spiked swabs. J Clin Microbiol.

[10] Dauphin LA, Moser BD, Bowen MD (2009). Evaluation of five commercial nucleic acid extraction kits for their ability to inactivate Bacillus anthracis spores and comparison of DNA yields from spores and spiked environmental samples. J Microbiol Methods.

[11] Kramvis A, Bukofzer S, Kew MC (1996). Comparison of hepatitis B virus DNA extractions from serum by the QIAamp blood kit, GeneReleaser, and the phenol-chloroform method. J Clin Microbiol.

[12] Schuurman T, van Breda A, de Boer R, Kooistra-Smid M, Beld M, Savelkoul P, Boom R (2005). Reduced PCR sensitivity due to impaired DNA recovery with the MagNA Pure LC total nucleic acid isolation kit. J Clin Microbiol.

[13] Zhang Z, Kermekchiev MB, Barnes WM (2010). Direct DNA amplification from crude clinical samples using a PCR enhancer cocktail and novel mutants of Taq. J Mol Diagn.

[14] Kermekchiev MB, Kirilova LI, Vail EE, Barnes WM (2009). Mutants of Taq DNA polymerase resistant to PCR inhibitors allow DNA amplification from whole blood and crude soil samples. Nucleic Acids Res.

[15] Schrader C, Schielke A, Ellerbroek L, Johne R (2012). PCR inhibitors - occurrence, properties and removal. J Appl Microbiol.

[16] Wang Y, Prosen DE, Mei L, Sullivan JCM, Vander Horn PB (2004). A novel strategy to engineer DNA polymerases for enhanced processivity and improved performance in vitro. Nucleic Acids Res.

[17] Loh E, Choe J, Loeb LA (2007). Highly tolerated amino acid substitutions increase the fidelity of Escherichia coli DNA polymerase I. J Biol Chem.

[18] Aboud M, Oh HH, McCord B (2013). Rapid direct PCR for forensic genotyping in under 25 min. Electrophoresis.

[19] Kamsteeg EJ, Kress W, Catalli C, Hertz JM, Witsch-Baumgartner M, Buckley MF, van Engelen BG, Schwartz M, Scheffer H (2012). Best practice guidelines and recommendations on the molecular diagnosis of myotonic dystrophy types 1 and 2. Eur J Hum Genet.

[20] Brook JD, McCurrach ME, Harley HG, Buckler AJ, Church D, Aburatani H, Hunter K, Stanton VP, Thirion JP, Hudson T (1992). Molecular basis of myotonic dystrophy: expansion of a trinucleotide (CTG) repeat at the 3′ end of a transcript encoding a protein kinase family member. Cell.

[21] Prior TW, American College of Medical Genetics Laboratory Quality Assurance C (2009). Technical standards and guidelines for myotonic dystrophy type 1 testing. Genet Med.

